# Reprogramming the tumor microenvironment – macrophages emerge as key players in breast cancer immunotherapy

**DOI:** 10.3389/fimmu.2024.1457491

**Published:** 2024-11-26

**Authors:** Ana Sami, Afsheen Raza

**Affiliations:** ^1^ Department of Medicine and Dentistry, Barts Cancer Institute, Queen Mary University of London, London, United Kingdom; ^2^ Department of Biomedical Sciences, College of Health Sciences, Abu Dhabi University, Abu Dhabi, United Arab Emirates

**Keywords:** immunotherapy, breast cancer, tumor microenvironment, tumor-associated macrophages (TAMs), nanoparticle, epigenetics

## Abstract

Breast cancer has the highest global incidence among all cancers, affecting more than 2 million individuals annually. Despite the availability of new drugs and novel treatment combinations, it is postulated that the incidence and mortality of breast cancer will rise by 40.8% and 51.9% respectively by 2040. Such dire statistics are associated with the clonal evolution of cancer cells that leads to therapeutic resistance and consequent relapse in breast cancer patients. On the other hand, the tumor microenvironment (TME) comprising of tumor cells, cancer-associated immune cells, re-programmed stromal cells, and the extracellular matrix (ECM) creates an immunosuppressive niche facilitating immune evasion. This review focuses on a critical cellular component of the tumor microenvironment, the tumor-associated macrophages (TAMs) in breast cancer immunotherapy. Macrophages are inherently plastic and can convert from an anti-tumor M1 phenotype to a pro-tumor M2 phenotype based on microenvironmental cues. Cancer cells facilitate these cues, allowing the tumor-associated macrophages to gain M2 phenotype and mediate immune evasion. Therefore, knowledge of the distinct role of tumor-associated macrophages in immune evasion can help design therapeutics such as engineered macrophages, M2 targeting drugs, and novel macrophage-mediated drug delivery strategies for long-term survival in breast cancer.

## 1 Introduction

Breast Cancer (BC) is a leading cause of morbidity and mortality in females affecting more than 2 million individuals annually (1). This is due to multiple factors, the most important being tumoral heterogeneity.

Breast cancer heterogeneity, facilitated by clonal evolution of the tumor or by cancer stem cells (2) is associated with therapeutics that lead to the selection and propagation of drug-resistant clones, causing resistance to treatment modalities (3–5). At least 20% of patients receiving treatment for breast cancer relapse within 5 years, resulting in metastatic disease with poor outcomes (6, 7).

To facilitate cancer progression and resistance to treatment modalities, tumor cells interact with various cellular components within the microenvironment to create a highly favorable tumor microenvironment (TME). The TME components consist of tumor cells, immune cells, re-programmed stromal cells, and immunosuppressive cells such as the Myeloid-Derived Suppressor Cells (MDSCs), regulatory T cells (Tregs), Tumor-Associated Macrophages (TAMs), cytokines and chemokines, etc. These components not only facilitate the generation of a hypoxic environment and epithelial to mesenchymal transition (EMT), they also play a significant role in the suppression of innate immune response and exhaustion of cytotoxic T and Natural Killer (NK) cells (8), thus promoting a vicious cycle that facilitates uncontrolled proliferation and tumor growth (9–11).

Though all components of the TME impact immune evasion and therapeutic resistance, tumor-associated macrophages are key modulators of these events. This is due to their ability to undergo cellular plasticity that catalyzes their phenotypic change from M1 pro-inflammatory to M2 anti-inflammatory phenotype in response to external and internal stimuli (12). In the TME, the dynamic interaction between the tumor cells and macrophages stimulates cellular plasticity and the M2 phenotype. M2 macrophages or TAMs, due to their immunosuppressive nature, promote drug resistance, making these cells attractive targets for cancer therapeutics (9, 13).

In this review, we aim to focus on the impact of TAM cellular plasticity on breast cancer immunotherapy. Novel approaches to TAM targeting that enhance immunotherapy efficacy and counter therapeutic resistance have been discussed to provide a broad understanding of the role of TAMs in improving patient outcomes in breast cancer.

### 1.1 Classically activated macrophages (M-1 phenotype)

Macrophages activate and modulate the innate immune response through phagocytosis, cellular cytotoxicity, secretion of pro-inflammatory cytokines such as Interleukin-6 (IL-6), IL-12, tumor necrosis factor (TNF), and antigen presentation to cytotoxic T cells through the major histocompatibility complex II (MHC-II) molecules (14). Their phenotype is significantly influenced by the tumors they infiltrate (15). In breast cancer, macrophages constitute greater than 50% of the tumor-infiltrating cells indicating their significance in the pathogenesis of BC (16).

Macrophages are classified into the M1-anti-tumor phenotype and the M2-pro-tumor phenotype (17). M1 Macrophages overexpress cell surface markers CD80, CD86, and CD16/32 (18) as shown in **Supplementary Figure 1**, leading to the activation of T cells. Furthermore, signaling through Toll-like receptors (TLRs) in M1 macrophages stimulates the activation of various transcription factors such as nuclear factor kappa-light-chain-enhancer (NF-κB) which in turn initiates the production of pro-inflammatory cytokines such as Interferon-γ (IFN-γ) (19, 20). In addition to this, the IFN-γ produced by T Helper Cell 1 (Th1) promotes M1 macrophage polarization and upregulation of nitric oxide synthase-2 (NOS2) production (21). NOS2 is a major activator of cytotoxic activity against tumor cells via oxidation of L-arginine to L-citrulline and cytotoxic nitric oxide (22, 23). The role of M1 macrophages in eradicating tumor cell proliferation and growth in BC has been evidenced in several studies. For example, a survey of forty HER2+ breast cancer patients, treated with trastuzumab reported that patients with elevated levels of M1-like macrophages (iNOS+) exhibited significantly improved survival. In contrast, high levels of M2-like macrophages (CD163+) were associated with poor prognosis (24).

### 1.2 M2 phenotype or tumor-associated macrophages

Pro-tumor macrophages or anti-inflammatory Tumor-Associated Macrophages (TAMs) are derived from circulating monocytes and tissue-resident macrophages. Tumor cells initiate recruitment of monocyte-derived circulating macrophages by production of Colony Stimulating Factor 1 (CSF1) and CC chemokine Ligand 2 (CCL2), leading to their binding to CSF1 receptor (CSF1R) and CCL2 receptor (CCR2) on the macrophage cell surface. This binding initiates the conversion of M1 anti-tumoral macrophage into a tumor-associated M2 macrophage (25, 26). On the other hand, Tissue-resident macrophages (TRMs), upon tumoral stimulation, activate Tregs thus initiating epithelial-mesenchymal transition (EMT), tumor invasion, and metastasis via the TAM phenotype (27).

Bone-marrow-derived monocytes can also convert to M2 phenotype when stimulated with immunosuppressive cytokines such as IL-10, or TGF-β in the TME, via phosphatidylinositol 3 kinase (PI3K)/protein kinase B (Akt) signaling pathway (28, 29). Studies on mouse models of breast cancer have documented that IL-10 secreted by TAMs prevents CD8+ T cell cytotoxic activity by inhibiting IL-12 expression on dendritic cells required for anti-tumoral differentiation of T cells into T helper-1 (Th1) cells (30). Moreover, IL-4 and IL-13 secreted by M2 macrophages stimulate T helper-2 (Th2) to secrete anti-inflammatory cytokines and chemokines that inhibit M1 polarization (31), and promote tumor cell growth and proliferation.

Hypoxia within the tumor microenvironment induces the TAM/M2 phenotype. Rapid tumor growth leads to hypoxic conditions and lactic acid accumulation within the TME leading to Vascular Endothelial Growth Factor (VEGF) and Arginase I (ARG-1) expression by macrophages (32). VEGF is a critical factor associated with the promotion of tumor angiogenesis and support for sustained tumor growth. On the other hand, ARG-1 hydrolyzes L-arginine (an essential amino acid required for T-cells and NK cell activation) to urea and L-ornithine (**Supplementary Figure 1**) leading to the inhibition of T and NK cell activation and proliferation (33, 34)

Studies on pre-clinical BC models have reported that Granulocyte-macrophage colony-stimulating factor (GM-CSF) secreted by tumor cells promotes programmed death ligand-1 (PD-L1) overexpression on TAM cell surface, thereby deactivating CD8^+^ cytotoxic T cells via the immunosuppressive PD-1/PD L1 interaction (35). Thus, it is evident that TAMs contribute, via multiple mechanisms, to promoting the uncontrolled proliferation of tumor cells and supporting immune evasion, leading to the invasion and metastasis of breast cancer.

### 1.3 TAM-targeted therapies for breast cancer

Strategies to reduce M2-macrophages in breast cancer tumor microenvironment include inhibition of macrophage recruitment, TAM depletion, and repolarization of pro-tumoral M2- -macrophages to the anti-tumoral M1 phenotype. Keeping these strategies in perspective, several novel TAM-targeting drugs are being tested in clinical trials and pre-clinical studies to improve the efficacy of BC immunotherapy, as shown in **Table 1**. Some of these drugs are discussed below:

**Table 1:** Pre-clinical studies and clinical trials targeting TAMs in breast cancer.

No.	Drug	Target	Target Population	Pre-clinical Model/Clinical Trial Phase	NCT	Observation	Reference
**1**	Pexidartinib (PLX3397) + Paclitaxel	CSF-1R	Transgenic and syngeneic mouse models **+** human BC cell lines	MMTV-PyMT mice **+** Cell lines: BT474, MDA-MB-435, SKBR3, T47D, MCF7, and MDA-MB-231		↓ TAMs ↑ CD8+ T cells	(36)
	Pexidartinib (PLX3397) + Eribulin	CSF-1R	Metastatic BC and TNBC patients	Phase Ib/2	NCT01596751	Phase I: 16.7% DLTs Phase II: 37.5% PFS, 16% OR	(37)
**2**	Tinengotinib (TT-00420)	Multi-kinase inhibitor	Human TNBC cell line + Syngeneic TNBC mouse models + xenograft model	HCC1806 + 4TI tumors in Balb/c mice + patient-derived xenografts		↓ TAMs ↑ T-cells	(38)
	Tinengotinib (TT-00420)	Multi-kinase inhibitor	Solid tumors inc. TNBC patients	Phase I	NCT03654547	16.7% PR 52.4% SD	(39)
	Tinengotinib (TT-00420)	Multi-kinase inhibitor	PC, HR+/HER2- BC, TNBC, CCA.	Phase Ib/II	NCT04742959	70.5% G1-3 TRAEs 50% ORR (TNBC) 60% DCR	(40)
**3**	Magrolimab (Hu5F9-G4) **+** Trastuzumab	CD-47 + HER2	Human HER2+ BC cell lines	SKBR3 and BT474		↑ ADCP	(41)
	D3L-001 (Bispecific antibody) **+** paclitaxel	CD-47 + HER2	Human BC cell lines and xenograft models	HCC1954, JIMT-1. Patient-sourced xenograft		↑ ADCP	(42)
	Magrolimab (Hu5F9-G4) + nab-paclitaxel/SG	CD-47	mTNBC patients	Phase II - ELEVATE TNBC	NCT04958785	Results not published	(43)
**4**	Anti-FBG + anti-PDL-1	Tenascin-C	Mouse models of BC	Orthotopic and autochthonous mammary tumors		↓ M2 polarization ↑ CD8+ T-cells ↓ tumor volume	(44)
**5**	IPI-549	PI3Kγ	Syngeneic mouse models of BC	PyMT+ metastatic BC		M2 ➔ M1 ↑ NFκB ↓ PD-L1	(45)
	IPI-549 + Atezolizumab + nab-paclitaxel	PI3K + anti-PD-L1 + microtubules	Advanced/Metastatic TNBC	Phase II -(MARIO-3)	NCT03961698	Results are awaited	(46)
**6**	AS1517499 (AS) + siRNA	STAT-6 + IKKβ	Mouse models of BC	Orthotopic tumors		M2 ➔ M1 ↓ side effects ↓ tumor growth & metastases	(47)
**7**	Resiquimod LNP	TLR7/8	Mouse models of BC	Orthotopic tumors		↑ M1 ↑ T-cells	(48)
**8**	DTX-M1-Exo	M2 macrophages and breast cancer	Mouse models of BC	4TI and RAW264.7 murine cell lines		M0 ➔ M1	(49)
**9**	OX40L M1 Exo	M2 macrophages	Mouse models of BC	BALB/c mice		M2 ➔ M1 Activate T cells ↑ IFNγ	(50)
**10**	Ec-PR848 (*E.coli* MG1655 + PDox+PR848)	M2 macrophages and breast cancer	Orthotopic model of BC	4TI cells in BALB/c mice		↑ ICD M0 ➔ M1	(51)
**11**	DPLGA@[RAW-4T1] NPs	Breast cancer lung metastasis	Mouse models of breast cancer lung metastasis	Fused 4TI and RAW264.7 cancer-macrophage hybrid membrane		88.9% anti-metastasis efficacy	(52)
**12**	CD147 CAR-M	CD147	Human breast cancer cell line	MDA-MB-231		↑ Anti-tumor activity ↓ Systemic toxicity	(53)
**13**	VEGFR2 CAR-M	VEGFR2	Mouse models of breast cancer	4TI cell line		M0 ➔ M1 ↓ Tumor growth	(54)
**14**	CT-0508	Anti-HER2 CAR-M	HER2+ solid tumor patients	Phase I	NCT04660929	37.5% SD, 12.5% PD	(55)
**15**	EZH2 knockdown	miR-124-3p ➔ inhibits CCL2	Human breast cancer cell lines	MDA-MB-231, MCF7, and BT549.		↓ TAM recruitment ↓ M2 polarization	(20)
**16**	Phenelzine	LSD1-CoREST complex	Mouse models of BC	TNBC		↑ M1 ↑ CD8+ T cells	(56)
	Phenelzine + nab- paclitaxel	LSD1-CoREST complex + microtubules	Advanced/Metastatic BC	Phase-I EPI-PRIMED	NCT03505528	PFS = 34wks RP2D =60mg	(57)
**17**	Tucidinostat	HDAC	Mouse model of BC	Syngeneic mouse model of breast, lung and colorectal cancer		M2 ➔ M1 ↑ NFκB ↑ CCL5	(58)
	Tucidinostat + Exemestane	HDAC + Aromatase inhibitor	Advanced ER+ BC	Phase-3	NCT02482753	PFS = 7.4 months ↑ TrAEs	(59)

DLT, Dose Limiting Toxicities; PFS, Progression-free-survival; OS, Overall survival; PD, Progressive Disease; SD, Stable Disease; R/R, Relapsed/Refractory; PR, Partial Response; OR, Objective Response; CR, Complete Response; ICD, Immunologic Cell Death; ADCP, Antigen Dependent Cellular Phagocytosis; PC, Prostate Cancer; CAA, Cholangiocarcinoma; TRAEs, Treatment-Related Adverse Events; ORR, Overall Response Rate; DCR, Disease Control Rate; G, Grade; RP2D, Recommended Phase 2 Dose; LNP, Lignin Nanoparticle.

#### 1.3.1 Pexidartinib; anti-Colony Stimulating Factor-1 Receptor

**Figure 1:**
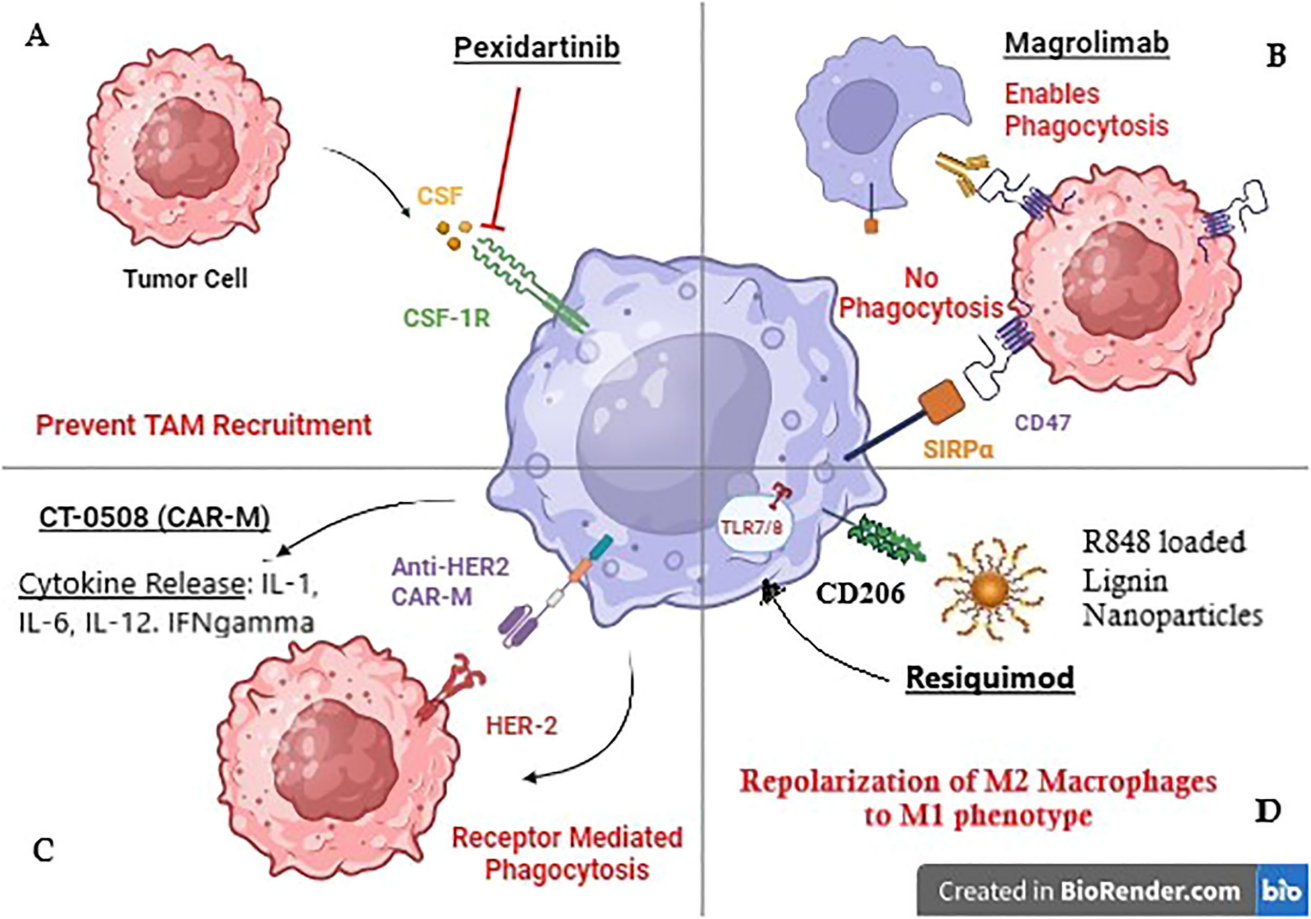
Mechanism of action of TAM-targeted therapies in Breast cancer. **(A)** Pexidartinib inhibits CSF-1R receptor on TAMS to prevent their recruitment to the tumor site. **(B)** Magrolimab blocks CD47 receptor on tumor cells to prevent its binding with Macrophage receptor SIRPa promoting phagocytosis. **(C)** CT-0508 is a HER-2 targeting CAR-M that has shown promising results in clinical trials. **(D)** Resiquimod is an R848 (TLR 7/8 agonist) loaded lignin nanoparticle targeting CD206 Mannose receptor on macrophages.

Pexidartinib (PLX3397) is an oral, small-molecule inhibitor of Colony Stimulating Factor-1 Receptor (CSF1R). CSF1R is implicated in the recruitment of macrophages to the TME, and their polarization to an anti-tumor M2 phenotype. Mechanistically, CSF-1 produced by tumor cells, binds to CSF1-receptor (CSF1-R) on the macrophage cell surface as shown in **Figure 1** (25). This binding alters macrophage metabolism via the downstream PI3K/Akt signaling pathway and promotes the expression of rapamycin complex -2 (mTORC-2), and interferon regulatory factor 4 (IRF4), leading to M2 macrophage polarization (60). Therefore, Pexidartinib, a CSF1-R inhibitor, reduces TAM recruitment within the TME and enhances the cytotoxic activity of immune cells.

In pre-clinical mouse models, Pexidartinib has been shown to revert immune suppression, reduce tumor growth, and improve survival (36). A Phase Ib/II Clinical trial (NCT01596751) tested Pexidartinib (PLX3397) in combination with non-taxane chemotherapy Eribulin in metastatic breast cancer patients. Promising results from Phase Ib reported dose-limiting toxicities (at 800mg/day and 1000mg/day dose) in 16.7% of patients, while Phase II results showed 3 months of progression-free survival in 35.7% of TNBC patients and objective response in 16% of patients (61).

#### 1.3.2 Tinengotinib (multi-kinase inhibitor)

Tinengotinib (TT-00420) is an oral, multi-kinase inhibitor that targets kinases Aurora A/B, Fibroblast Growth Factor Receptor (FGFR1/2/3), Vascular Endothelial Growth Factor Receptors (VEGFRs), Janus Kinases (JAK1/2), and CSF1R (38).

In pre-clinical studies on syngeneic mouse models of TNBC, Tinengotinib treatment resulted in up-regulation of chemokines CXCL10 and 11 (ligands for T-cell receptor CXCR3) leading to increased infiltration of T cells and reduced numbers of tumor-associated macrophages (TAMs). Inhibition of tumor proliferation, decreased angiogenesis, and an amplified immune response were observed in this study (38). The study provided evidence for a *‘first-in-human’* phase I clinical trial of Tinengotinib, for treating TNBC (NCT03654547) (62). Promising results were reported with 7/42 (16.7%) patients exhibiting partial response (PR) and 22/42 (52.4%) showing stable disease (SD). Additionally, the drug was well tolerated with a limited number of patients experiencing dose-limiting toxicities (39).

#### 1.3.3 Magrolimab (Anti-CD47)

Tumor cell surface molecule CD47 binds to its ligand, the signal regulatory protein α (SIRPα) on the macrophage cell surface, and provides a safety signal, blocking phagocytosis (63). CD47 is overexpressed in HER2+ tumors. Macrophage checkpoint blockade, using the anti-CD47 antibody Magrolimab has been investigated in HER2+ breast cancer cells in combination with the anti-HER2 antibody Trastuzumab (41). Results showed upregulation of macrophage-mediated, Fc-dependent antibody-dependent cellular phagocytosis (ADCP).

A phase II clinical trial ELEVATE TNBC (NCT04958785) is underway in patients with locally advanced or metastatic TNBC. The trial combines CD47 inhibition through Magrolimab (Hu5F9-G4) with taxane chemotherapy nab-paclitaxel/paclitaxel or sacituzumab govitecan-hziy (SG) (44, 49, 52). SG is an antibody-drug conjugate that combines the anti-trophoblast cell-surface antigen 2 (Trop-2) antibody hRS7 IgG1, with a topoisomerase1 inhibitor SN-38, inhibiting DNA replication. Trop-2 is overexpressed in up to 90% of TNBC with poor prognoses (64, 65). The trial aims to investigate the potential of macrophage-mediated ADCP due to the amplification of phagocytic signals on the tumor cell surface after taxane treatment (43).

D3L-001, a bispecific antibody against HER2 and CD47 was investigated in HER-2+ breast cancer cell lines HCC1954, JIMT-1, and patient-sourced xenograft models of breast cancer. Results showed significant anti-tumor activity due to the synergistic effect of simultaneous dual targeting of HER2 and CD47 leading to antibody-dependent cellular phagocytosis. D3L-001 also showed improved efficacy when combined with chemotherapy such as paclitaxel (42).

#### 1.3.4 Anti-FBG (Fibrinogen-like globe) antibody

Tenascin-C (TNC) is an extracellular matrix (ECM) glycoprotein, overexpressed in solid tumors. Tumor-derived Tenascin-C facilitates immunosuppression and promotes pro-tumor (M2) macrophage phenotype via activation of macrophage toll-like receptor-4 (TLR-4) through the binding of the TNC protein C-terminal Fibrinogen-like globe (FBG) domain (66). This is a paradoxical situation as TLR-4 classically activates the pro-inflammatory M1 macrophage phenotype.

Pre-clinical studies on murine models of breast cancer have documented that, anti-FGB treatment, when combined with anti-PDL-1 immunotherapy, led to reduced polarization of macrophages to TAMs, increased infiltration of CD8+ cytotoxic T lymphocytes, and significant reduction of tumor volume and lung metastases (44).

### 1.4 Macrophage re-polarization therapy

Macrophages constitute 50% of the cells infiltrating the TME, thus repolarizing anti-inflammatory M2 TAMs to pro-inflammatory M1 phenotype can greatly turn the odds in our favor (16). Multiple strategies have been evaluated in this regard, including but not limited to, Phosphatidylinositol 3-kinases (PI3Ks) inhibitors, STAT inhibitors, and Toll-like receptors agonists (TLR-3/4/7&8/9 agonists) (67).

PI3Ks promote cell survival, proliferation, and differentiation (68). PI3Kγ, via Akt and mTOR signaling, blocks NF-κB activation, and stimulates transcription factor CCAAT/enhancer binding protein β (C/EBPβ). C/EBPβ, in turn, promotes the M2-associated genes (including Arg1, Il10, and Mrc1), causing immunosuppression (69, 70). In syngeneic mouse models of breast cancer, PI3K-γ inhibitor IPI-549 repolarized TAMs from M2 to M1, stimulated macrophage NF-κB expression, and reduced PD-L1 expression, thus promoting CD8 + T cell cytotoxicity (45). A phase II clinical trial MARIO-3 (Macrophage Reprogramming in Immuno-Oncology) (NCT03961698) is currently underway to evaluate IPI-549 in combination with Atezolizumab and nab-paclitaxel as first-line therapy in advanced/metastatic TNBC patients. The results of the trial are awaited (46).

The JAK/STAT signaling pathway is integral to M1/M2 macrophage polarization (69). The STAT6 transcription factor regulates genes such as arginase 1 (Arg1), and macrophage mannose receptor 1 (Mrc1) required for IL-4-mediated M2 macrophage polarization (71). Transcription factor NF-κB activates a pro-inflammatory TME (72). Upstream inhibitors of NF-κB activation, Inhibitory kappa B kinases (IKKs), prevent inflammatory response and M1 macrophage polarization (73). An M2 macrophage targeting, Ph-sensitive, PEG-coated nanodrug encapsulating STAT6 inhibitor AS1517499 (AS) and IKKβ siRNA was tested in orthotopic mouse models of breast cancer. The therapy reduced tumor growth and metastasis and caused M2 to M1 macrophage polarization. Targeted drug delivery to TAMs also reduced immune side effects (47).

Toll-like receptors (TLRs) are transmembrane or intracellular pattern recognition receptors on antigen-presenting cells such as macrophages (74). Endosomal TLR 7/8 recognizes single-stranded RNA to initiate an inflammatory response (75). In pre-clinical orthotopic mouse models of TNBC, TLR 7/8 agonist Resiquimod was tested through lignin nanoparticles (LNPs). These LNPs targeted CD206+ M2 TAMs via the mUNO peptide, reducing off-target effects, as shown in **Figure 1**. Results showed increased numbers of M1 macrophages and cytotoxic T cells. The therapy had a synergistic effect with chemotherapy Vinblastine (48).

These studies testify to the impact of Macrophage polarization on tumor growth, proliferation, and drug resistance.

### 1.5 Engineered macrophages for drug delivery

Macrophages are the most abundant immune cells in the tumor microenvironment, making them attractive agents for drug delivery. They also have a prolonged half-life and an inherent ability to migrate to the tissue microenvironment, thus reducing off-target effects and associated toxicities. The applications of engineered macrophages in cancer immunotherapy include macrophage-mediated drug delivery, chimeric antigen receptor macrophage (CAR-M) therapy, and combined treatment approaches deploying bacterial drug delivery and macrophage exosomes (76).

#### 1.5.1 Docetaxel-M1; DTX-M1-exosomes

Immune cell-derived exosomes can be used as vehicles to deliver chemotherapeutic drugs such as docetaxel (DTX). A pre-clinical study investigated anti-tumoral M1 macrophage-derived exosomes (M1-Exo) loaded with Docetaxel to create the DTX-M1-Exo drug delivery system. The experiment utilized the 4T1 murine breast cancer cell line and RAW264.7 murine macrophage cell line for *in-vivo* mouse models of breast cancer. The therapy induced polarization of M0 macrophages to the M1 phenotype in the immunosuppressive TME, while inhibiting repolarization to the pro-tumoral M2 phenotype. Thus, DTX-M1-Exo is a novel treatment combining immunotherapy through macrophage polarization with docetaxel chemotherapy to achieve substantial antitumor therapeutic efficacy (49).

#### 1.5.2 OX40L M1 exosomes

The OX40 receptor is expressed on the surface of immune cells, such as CD8^+^ T cells while its ligand OX40L is expressed on antigen-presenting cells, such as macrophages (77). In an *in-vivo* study conducted on mouse models of breast cancer, OX40L M1-exosomes accumulated in tumor tissues, repolarized M2-macrophages into M1-macrophages and promoted phagocytosis. OX40L M1-exos activated T-cells by binding to OX40 on their cell surface, promoting IFN-γ secretion. Thus, the dual impact of innate and adaptive immunity successfully blocked the growth and metastasis of mouse breast cancer (50).

#### 1.5.3 Bacterial therapy

Bacteria have tumor-homing ability and can survive in hypoxic conditions in the TME. They are thus being deployed as therapeutic carriers for anti-tumor drugs. Ec-PR848 comprises *E.coli* MG1655 loaded with 2 types of nanoparticles. The PDOX nanoparticles (NP) consist of chemotherapy doxorubicin (Dox)-loaded onto poly lactic-co-glycolic acid (PLGA) core, while PR848 consists of toll-like receptor 7/8 (TLR7/8) agonist Resiquimod (R848) loaded onto PLGA. R8484 binds to macrophage endosomes to promote an anti-tumor M1 phenotype (51). This combines macrophage re-polarization therapy with chemotherapy-induced immunologic cell death (ICD), which activates cytotoxic T cells and promotes their infiltration into the TME.

#### 1.5.4 Cell membrane-based nanoparticles

In a pre-clinical study on mouse models of breast cancer lung metastasis, cell membranes from the mouse macrophage RAW264.7 cells and mouse breast cancer 4T1 cells were fused to create a macrophage-cancer hybrid membrane. This was then coated onto doxorubicin (Dox)-loaded poly (lactic-co-glycolic acid) (PLGA) NPs (DPLGA@[RAW-4T1] NPs) for treating breast cancer lung metastasis *in-vivo*. The results exhibited 88.9% anti-metastasis efficacy due to metastasis targeting through the α4 and β1 integrin expression, on the macrophage membrane thus showing potential as a future drug delivery strategy in metastatic disease (52).

#### 1.5.5 CAR-M cellular therapy

Adoptive Cellular therapy utilizing Chimeric Antigen Receptor T cells (CAR-T) cells has evolved to incorporate genetically engineered Macrophages (CAR-Ms). CAR-Ms offer the potential benefit of producing Matrix Metalloproteases (MMPs) that degrade the ECM allowing greater access to the tumor site (78). This enables CAR-Ms to phagocytose tumor cells, present tumor antigens to T cells, and activate the TME as shown in **Supplementary Figure 2** (55). Macrophages express CD46, the docking protein for group B adenoviruses such as Ad35, making Adenoviruses reliable vectors for macrophage engineering (79).

CD-147 is overexpressed in multiple tumors including breast cancer. In a preclinical study on MDA-MB-231 cell lines CD147CAR-Ms were investigated. Enhanced antitumor activity and reduced systemic toxicity were observed indicating the role of CAR-M as a therapeutic tool to target cancer cells *in vitro* (53).

Vascular Endothelial Growth Factor Receptor (VEGFR2) is overexpressed in multiple tumors. Targeting of VEGFR2 via CAR-Ms was evaluated in 4T1 breast cancer mouse models. CAR-Ms were activated through TLR-4 and/or IFN-γ receptors leading to M1 macrophage polarization. This is due to the TLR intracellular domain of CAR-M. Enhanced macrophage infiltration into the tumors was observed resulting in reduced tumor size. This indicates the utility of CAR-Ms as inhibitors of BC growth and proliferation (54)

A *first-in-human* clinical trial (NCT04660929) is currently underway in HER2 overexpressing solid tumors including breast cancer. The trial is investigating CT-0508, an anti-HER2 CAR Macrophage, and Phase-1 results have shown that CT-0508 is well tolerated without any dose-limiting toxicities, adverse events, or on-target off-tumor activity. The trial reported 37.5% (3/8 patients) with stable disease and only 1 out of 8 patients exhibited progressive disease. Mechanistically, CT-0508 modulated the TME causing T cell activation, proliferation, and infiltration into the TME, making it a novel therapeutic agent in BC (55).

### 1.6 Epigenetic control of macrophage polarization

Epigenetic alterations induce phenotype changes by mediating the expression of transcription factors through histone modification, DNA methylation, histone acetylation and deacetylation, Micro RNA, etc.

These epigenetic alterations are also capable of influencing macrophage plasticity in the TME (76) due to their unique epigenomic memory leading to rapid phenotypic changes from recurrent environmental signals (80).

Enhancer of Zeste Homolog 2 (EZH2) causes histone H3 tri-methylation and is implicated in TAM polarization (81). Studies on CRISPR Cas9-mediated EZH2 knockdown in breast cancer cell lines MDA-MB-231, MCF7, and BT549, reported DNA demethylation and consequent upregulation of miR-124-3p and inhibition of its target gene CCL2, which helps recruit macrophages to the tumor site (20). This reduced TAM infiltration into the TME and inhibited M2 polarization (Qian et al., 2011).

Lysine-specific histone demethylase 1A (LSD1), is differentially expressed in M1 and M2 macrophages and is implicated in the epigenetic regulation of EMT, and cancer stem cell (CSC) genes (82). In murine models of TNBC, LSD1 inhibitor Phenelzine targets LSD1 binding domains nuclear REST corepressor 1 (CoREST) and flavin adenine dinucleotide (FAD) to disintegrate the LSD1-CoREST complex (56). This induces the expression of M1 macrophage genes, establishes the M1 phenotype, increases CD8+ T-cell infiltration into the TME, and improves the efficacy of anti-PD-1 immunotherapy (83, 84). A phase I clinical trial EPI-PRIMED (NCT03505528) evaluated the impact of Phenelzine Sulfate in combination with nab-paclitaxel in patients with advanced/metastatic breast cancer. Results showed median PFS at 34 weeks and established 60mg Phenelzine as the recommended Phase 2 dose (57).

Histone deacetylase (HDAC) is a molecular eraser that causes transcriptional repression (85). HDAC inhibitors stop cancer cell proliferation and upregulate MHC class I genes (86). In syngeneic murine breast, lung, and colorectal cancer models, HDAC inhibitor Tucidinostat polarized M2 macrophages to the M1 phenotype by activating the NF-κB signaling pathway, promoted the expression of chemoattractant CCL5 leading to the infiltration of CD8 + T cells, and decreased tumor resistance to anti-PD-L1 immunotherapy (58). A Phase 3 clinical trial NCT02482753 assessed the safety and efficacy of Tucidnostat (Chidamide) in combination with aromatase inhibitor Exemestane in patients with advanced Estrogen Receptor + (ER+) BC. Results showed improved PFS with a higher incidence of grade 3/4 hematological adverse events (59).

Alternatively, the transcription factor Zinc-finger E-box-binding homeobox 1 (ZEB1) facilitates EMT and is crucial for breast cancer invasion and metastasis. It causes the acetylation of DNA Methyl Transferase 1 (DNTM1), through histone acetyltransferase p300, resulting in increased expression. TAMs support DNMT1 overexpression through the IL-6-pSTAT3-ZEB1-DNMT1 pathway, that in turn supports breast cancer invasion (87). This elucidates the significance of epigenetics in promoting or halting cancer growth and metastasis.

#### 1.6.1 Micro RNA therapy

Micro RNAs are small non-coding RNAs implicated in macrophage activation, polarization, and cytokine secretion. MicroRNAs known to impact macrophage polarization include miR-147 and miR-223. These miRNAs when activated by TLR stimulation, downregulate macrophage production of pro-inflammatory cytokines IL-6 and IL-1β, indicating a pro-tumoral effect (88, 89). Similarly, miR-125a-5p when stimulated by TLR2/4, promotes M2 macrophage polarization via IL-4 and downregulates the M1 macrophage phenotype, (90) while miR-21 induces M2 macrophage polarization via blocking the JAK2/STAT1 signaling pathway (91). Knowledge of the critical signaling pathways associated with these micro-RNAs can facilitate miRNA-targeted drug development, and stimulate an anti-tumor TME for breast cancer immunotherapy.

For M1 macrophage polarization, miR-16 has been shown to influence polarization in mouse peritoneal macrophages via overexpression of M1 marker CD16/32, cytokine IL-12, and nitric oxide. Moreover, it also induces depletion of M2 marker CD206 indicating TAM inhibition. In addition to this, miR-16 also downregulates macrophage expression of PD-L1 (92), creating a conducive environment for immune cell infiltration. Similarly, miR-17, miR-20a, and miR-106a, when activated by lipopolysaccharide (LPS), have been reported to cause reduced signal regulatory protein α (SIRPα) expression, and promote pro-inflammatory cytokine secretion, (93) thus initiating an anti-tumor immune response.

## 2 Conclusion

Immunotherapy heralded as the miracle cure, has seen limited success in solid tumors, this is mainly due to the fibrotic and immunosuppressive TME. Utilizing immune cells abundantly found in the TME such as macrophages, has the potential to specifically target tumor cells, limiting systemic toxicities such as inflammation.

Therapies targeted towards the anti-tumor M2 Macrophages have shown positive results in pre-clinical studies. Deploying targeted therapies to revert the immunosuppressive nature of the TME can help eliminate tumors and prevent recurrence. Macrophage therapy despite showing promising results in pre-clinical studies, is still in its infancy and needs further characterization for safety and reliability. Emerging results from the *first-in-human* clinical trials shall light the path for the future of this innovative therapy.

Engineered macrophages hold great promise as drug delivery vehicles and as agents to influence an anti-tumor TME. Further research into lowering costs and creating a standardized version of this personalized therapy can help bring these therapies to the clinic and benefit patients with metastatic, relapsed/refractory breast tumors.

## Supplementary material

Supplementary Figure 1 Differences between M1 and M2 Macrophage Phenotype. Specific differences in activating molecules, cell surface receptors, secreted molecules, and Arginine metabolism.

Supplementary Figure 1 Properties of CAR-M cells. 1. CAR-M can recognize tumor-associated antigens (TAA) to initiate phagocytosis. 2. They secrete pro-inflammatory cytokines to facilitate immune cell infiltration. 3. They secrete Matrix Metalloproteinases (MMPs) to degrade extracellular matrix (EMC) and facilitate T-cell infiltration and drug access. 4. CAR-M present TAA to CD8+ T-cells via MHC-II molecules to activate cytotoxic immune response.
