# Supplementary figures and images for: Reprogramming the tumor microenvironment – macrophages emerge as key players in breast cancer immunotherapy
Source: Front Immunol. 2024 Nov 26;15:1457491. doi: 10.3389/fimmu.2024.1457491 (PMC11628348; doi:10.3389/fimmu.2024.1457491)

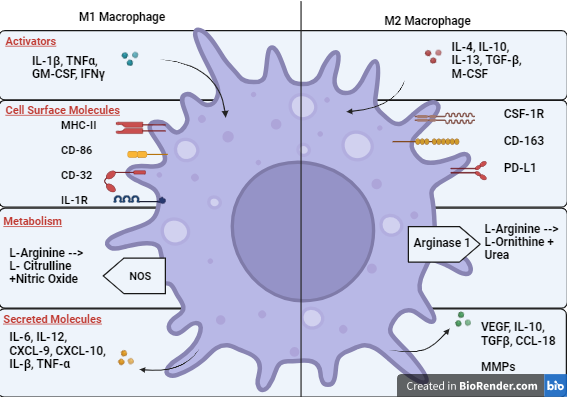

Supplement: Supplementary Figure 1 — Differences between M1 and M2 Macrophage Phenotype. Specific differences in activating molecules, cell surface receptors, secreted molecules, and Arginine metabolism. [file Image1.png]

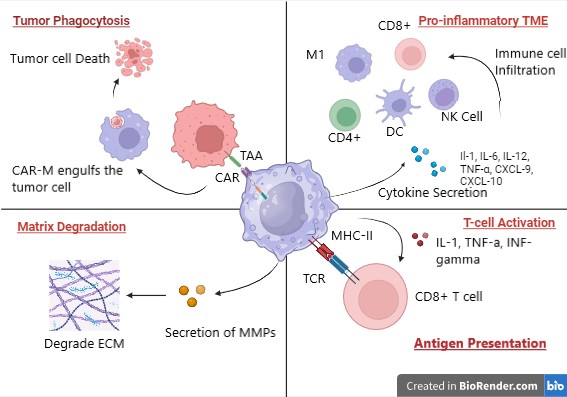

Supplement: Supplementary Figure 1 — Properties of CAR-M cells. 1. CAR-M can recognize tumor-associated antigens (TAA) to initiate phagocytosis. 2. They secrete pro-inflammatory cytokines to facilitate immune cell infiltration. 3. They secrete Matrix Metalloproteinases (MMPs) to degrade extracellular matrix (EMC) and facilitate T-cell infiltration and drug access. 4. CAR-M present TAA to CD8+ T-cells via MHC-II molecules to activate cytotoxic immune response. [file Image2.jpeg]
